# 5,10-Methylenetetrahydrofolate Reductase—the Key Allosteric Regulator in One-Carbon Metabolism

**DOI:** 10.1021/acs.biochem.5c00707

**Published:** 2026-02-27

**Authors:** Linnea K. M. Blomgren, Shuning Guo, D. Sean Froese, Thomas J. McCorvie, Wyatt W. Yue

**Affiliations:** † Division of Metabolism and Children’s Research Center, University Children’s Hospital Zürich, 27217 University of Zürich, Zürich CH-8008, Switzerland; ‡ Biosciences Institute, The Medical School, 5994 Newcastle University, Newcastle upon Tyne NE2 4HH, U.K.

## Abstract

Collectively known as one-carbon metabolism (OCM), both the folate and methionine cycles are highly regulated to meet cellular demands. These cycles are key in the production and recycling of methyl groups to be used in many essential cellular processes such as the production of nucleotides, as well as *S*-adenosyl-l-methionine (SAM) the global methyl donor for DNA, RNA, and post translational modifications. Within the folate cycle, 5,10-methylenetetrahydrofolate is the main species through which methyl groups enter OCM. Therefore, 5,10-methylenetetrahydrofolate reductase (MTHFR), which reduces 5,10-methylenetetrahydrofolate into 5-methyltetrahydrofolate, is the central enzyme that directs methyl groups for use within the methionine cycle. MTHFR is an enzyme found in all domains of life, but unlike in prokaryotes, eukaryotic MTHFR activity is highly regulated by the level of SAM, to balance the one-carbon needs of the cell. In this perspective, we review the catalytic mechanism of MTHFR, evolutionary differences, and the regulatory mechanisms that have evolved to alter its activity. We also discuss recent structural findings that reveal a unique mechanism for inactivation by SAM as a feedback loop and its consequences for understanding inherited MTHFR deficiency.

## Graphical abstract

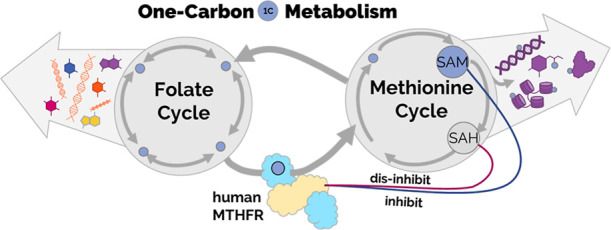

## Introduction: MTHFR at the Junction of Folate and One-Carbon Metabolism

The folate and methionine cycles are responsible for creating and regulating the building blocks of life through the interconversion of one-carbon units (Figure 1). In the folate cycle, one-carbon units generate purine and thymidine monophosphate to support the production and repair of DNA and RNA.^1,2^ One-carbon units may also be shuttled toward the methionine cycle, to generate the universal methyl donor *S*-adenosyl-l-methionine (SAM) for epigenetic modification and regulation of biomolecules.^3,4^ Situated at the juncture of these two cycles and regulating the metabolic flux of one-carbon units is the enzyme 5,10-methylenetetrahydrofolate reductase (MTHFR, EC1.5.1.20).

**Figure 1:**
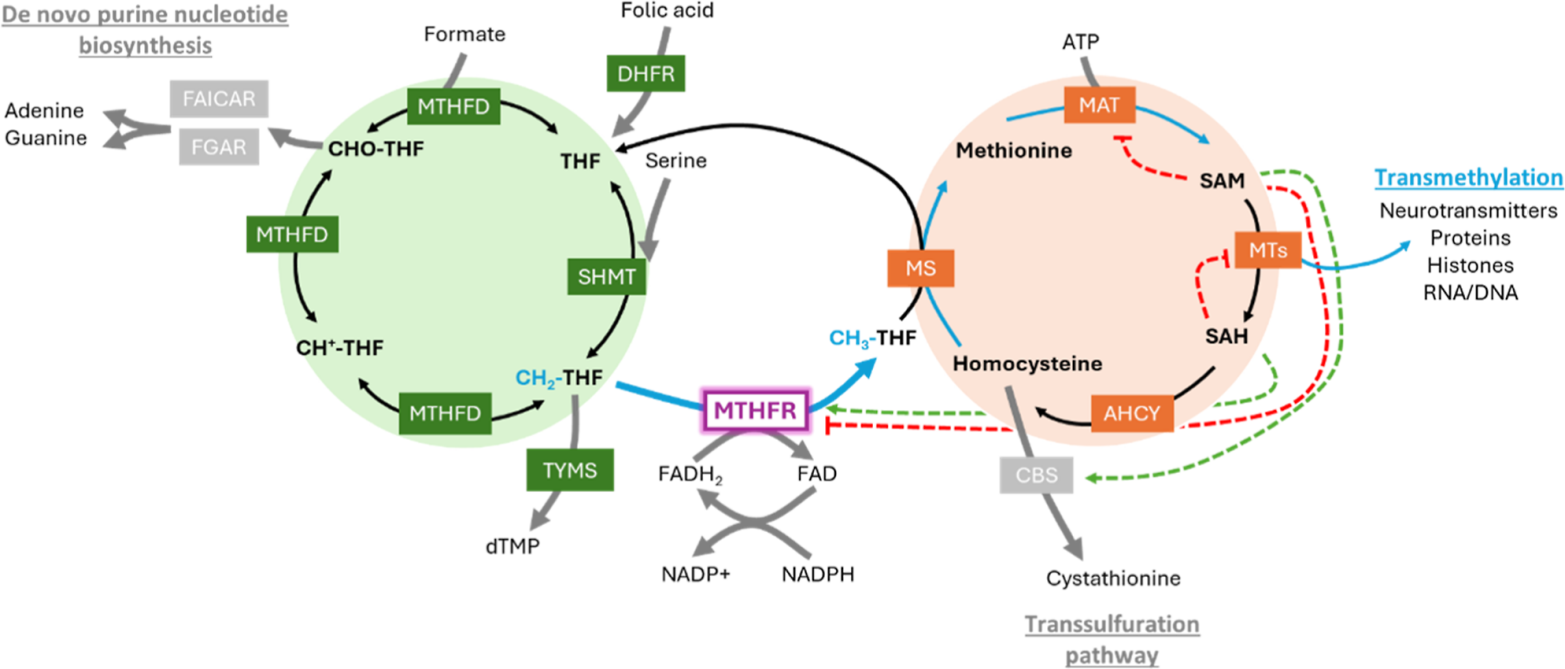
Schematic representation of OCM featuring the interconnectivity between folate cycle (green) and methionine cycle (orange). Metabolites in bold-type fonts, enzymes in colored boxes, feed-back regulation of SAM and SAH shown as red dashed lines for inhibition and green dashed lines for activation/disinhibition. Blue arrows and font indicate the unidirectional reaction of MTHFR that connects with SAM biosynthesis.

MTHFR catalyzes the physiologically irreversible reduction of the folate cycle intermediate 5,10-methylenetetrahydrofolate (CH_2_-THF), producing 5-methyltetrahydrofolate (CH_3_-THF). Since the product CH_3_-THF is exclusively used in the methionine cycle (by methionine synthase, MS, for the remethylation of homocysteine to generate methionine; Figure 1), and only the demethylated tetrahydrofolate (THF) will be recycled back to the folate cycle, MTHFR essentially shuttles one-carbon units away from nucleotide synthesis in the folate cycle and commits them to the methionine cycle for both methionine and SAM synthesis. This reaction is dependent on the reductive agent nicotinamide adenine dinucleotide phosphate (NADPH), whose main source is the pentose phosphate pathway.^5,6^ NADPH is also used in folate-dependent nucleotide synthesis, fatty acid, proline and deoxyribonucleotide synthesis, and reactive oxygen species defense.^5,7^ Therefore, to dedicate NADPH to MTHFR-catalyzed methionine and SAM synthesis, cells must have sufficient energetic capacity (in terms of ATP) and sufficient need (over and above the requirements for DNA synthesis and cell replication). Given this central role, and as a rate-limiting enzyme within one-carbon metabolism (OCM), dysregulation of MTHFR disrupts the flux between the two cycles with detrimental effects on human health. Both mild and severe forms of MTHFR deficiency manifest in a range of diseases, underlining the paramount importance and cellular dependence of MTHFR activity. It is small wonder that MTHFR has garnered so much scientific (>8000 publications in PubMed) and medical (described in >80 clinical trials) attention, underscoring its importance in metabolism and health.

Research on MTHFR biochemistry has been ongoing for more than 60 years. It began in the late 1950s and early 1960s when Donaldson and Keresztesy identified a CH_3_-THF-metabolizing enzyme in pig liver.^8,9^ Their discovery was followed up by Kutzbach and Stokstad in the early 1970s, showing porcine MTHFR to be a flavoprotein utilizing the cofactor flavin adenine dinucleotide (FAD) and the electron donor NADPH. It has also been long known that SAM allosterically inhibits MTHFR enzyme activity, a feedback regulation that can be reversed by *S*-adenosyl-l-homocysteine (SAH), the demethylated derivative of SAM,^10,11^ thus maintaining a fully active enzyme further fuelling the methionine cycle with one-carbon units.

In the past two decades, a flurry of structural studies of MTHFR has also taken off. Recent studies of the human enzyme have not only revealed a conserved catalytic machinery but also yielded unprecedented molecular insight into a eukaryote-specific regulatory mechanism involving a novel structure fold. In this perspective, we delve into the intricacy of MTHFR function and regulation through the eyes of structural biochemistry.

## MTHFR Catalysis Is Evolutionarily Conserved

MTHFR orthologues are widely distributed across all domains of life, with a highly conserved catalytic domain (CD) solely responsible for the enzymatic activity (Figure 2A).^11^ Acting as an oxidoreductase, MTHFR catalyzes the conversion of CH_2_-THF to CH_3_-THF, in the presence of the flavin cofactor FAD and dinucleotide electron donor NAD­(P)­H. As both substrates bind the same active site, MTHFR uses a ping-pong ordered Bi–Bi mechanism involving two hydride transfer steps.^12–14^ First, the incoming electron donor NAD­(P)H reduces the level of enzyme-bound FAD, generating NAD­(P)^+^ and FADH_2_. Second, the release of NAD­(P)^+^ makes way for the one-carbon substrate CH_2_-THF to be reduced by enzyme-bound FADH_2_ to form CH_3_-THF, restoring FAD to its oxidized state (Figure 2B).

**Figure 2:**
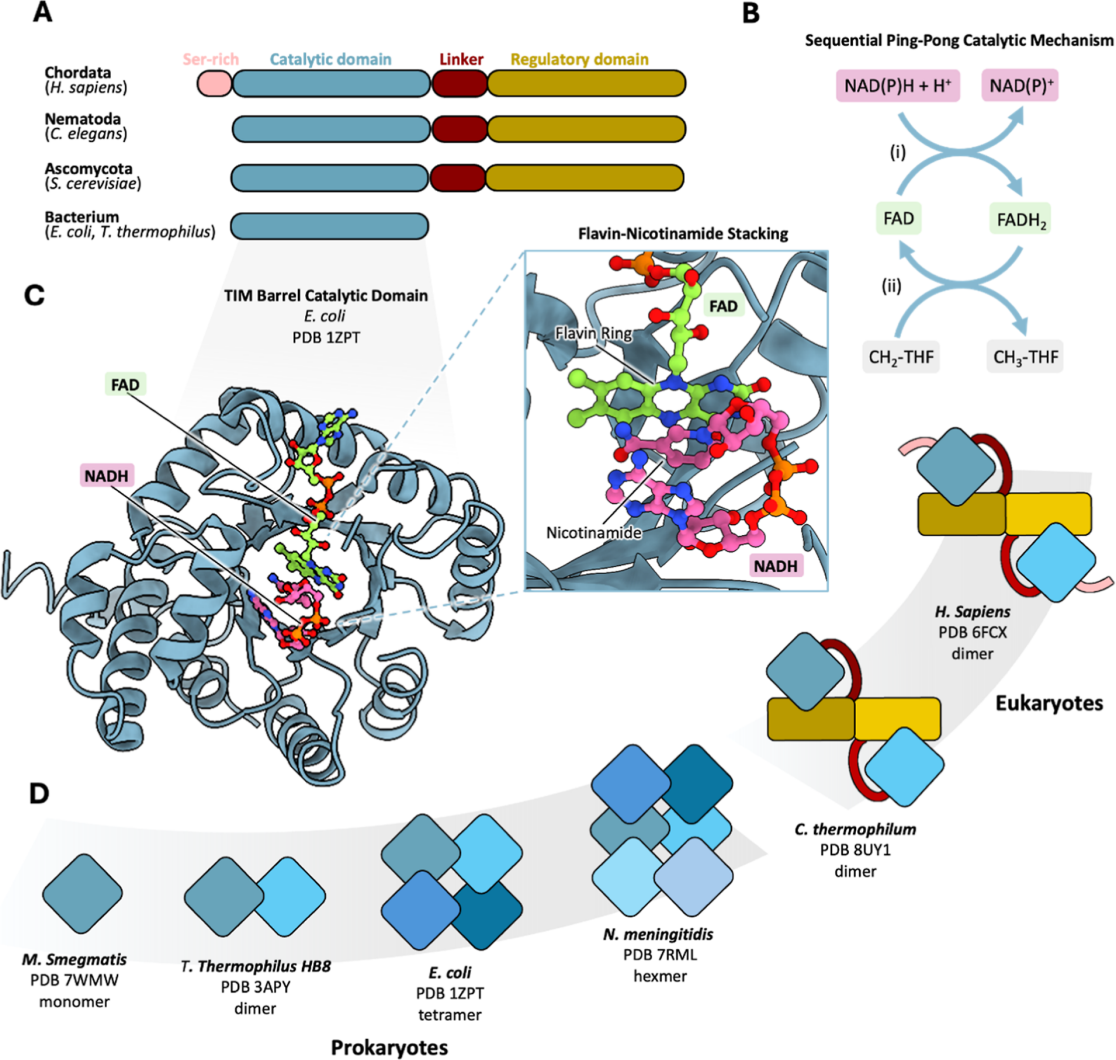
MTHFR across the kingdoms of life. (A) Domain organization of MTHFR orthologues across evolution revealing differences in the N- and C-termini. (B) Schematic depicting the ping-pong Bi–Bi mechanism of MTHFR. Step (i) begins with hydride transfer from NADH or NADPH to the FAD cofactor. The oxidized NAD^+^ or NADP^+^ then leaves the active site allowing step (ii) to occur. The reduced FAD then transfers hydrides to the substrate CH_2_-THF reducing it to CH_3_-THF. (C) Crystal structure of the CD of an *E. coli* MTHFR protomer, which reveals a TIM fold bound to the cofactor FAD (green) and the substrate NADH (pink). The inset shows the close interaction of the FAD and NADH where the flavin group of FAD stacks against the nicotinamide of NADH for hydride transfer. (D) Cartoon depiction of oligomeric states of MTHFR orthologues through available structures (PDB codes shown). While prokaryotic MTHFRs assemble into different oligomers through the CD (blue shades), eukaryotic MTHFR homodimerizes through its regulatory domain (RD) (yellow shades). Human MTHFR representative of Chordata differs from other eukaryotic MTHFR due to the presence of a Ser-rich region at its N-terminus.

Over the years, crystal structures of CD from different bacterial MTHFR orthologues (e.g., *Escherichia coli*, *Thermus thermophilus* *HB8*) have revealed a conserved TIM barrel fold, characterized by 8 alternating α-helices and β-sheet strands, with a tightly bound FAD nestled within its active site^15,16^ (Figure 2C). The active site configuration facilitates the binding of the substrate CH_2_-THF (through its pteridine ring) and electron donor NAD­(P)H (through its nicotinamide), in turn, to a shared site during the two-step reaction,^12,17,18^ to stack against the FAD flavin group for hydride transfer (Figure 2C). Bacterial MTHFRs show a preference for NADH as an electron donor,^19^ while mammalian MTHFRs have a strict preference for NADPH.^20,21^ Interestingly, MTHFR from plants, nematodes, and fungi exhibit varied preference for NADPH (e.g., *Saccharomyces cerevisiae* Met13) and NADH (e.g., *Arabidopsis thaliana* MTHFR).^22,23^ *Leishmania* MTHFR was reported to demonstrate dual cofactor specificity with NADH and NADPH, although its physiological significance is not clear.^24^

There are notable exceptions to this mechanism. In certain acetogenic anaerobic bacteria (e.g., *Acetobacterium woodii*, *Moorella thermoacetica*, *Clostridium ljungdahli*), their MTHFR enzymes (known as MetF) contain FMN, instead of FAD, as the flavin cofactor in the active site.^25–27^ These MetF proteins also do not bind the electron donor NADH.^28^ Instead, they encode accessory proteins which either harbor the NADH-binding sites (e.g., RnfC2 in *A. woodii* ^27^) or utilize another electron donor such as the iron–sulfur cluster (e.g., MetV in *C. ljungdahlii* ^28^), to work in concert with MetF.

Additionally, a group of flavin-independent mycobacterial MTHFRs (e.g., *Mycobacterium smegmatis* MSMEG_6649) have recently been shown to lack any flavin cofactor FAD or FMN at all.^29,30^ These enzymes likely catalyze a direct hydride transfer from NADH to CH_2_-THF, in a mechanism similar to the second step of the flavin-dependent enzymes. Indeed, the crystal structure of *holo* MSMEG_6649, when compared to *E. coli* MTHFR, revealed the position of NADH shifting into that of FAD while the CH_2_-THF-binding site appeared unchanged,^31^ suggesting that NADH and substrate can bind concurrently in a ternary complex for catalysis.

While MTHFR is a reversible enzyme *in vitro*, the forward reaction (CH_2_-THF to CH_3_-THF) is the physiologically relevant direction *in vivo* ^19,32^ due to the high cellular NADPH/NADP^+^ ratio (∼10^7^) in mammals^33^ and the large Gibbs free energy Δ*G* change for CH_2_-THF reduction.^34^ Plant MTHFRs (e.g., *A. thaliana*), however, are NADH-dependent, and because of a highly oxidized NADH/NAD pool (ratio of ∼10^–3^), they can catalyze the reaction in both directions. Recently, a subfamily of MTHFRs was identified, preferring to catalyze the reverse reaction, i.e., oxidation of CH_3_-THF to CH_2_-THF through reduction of FAD. An exemplar is the soil bacteria *Sphingobium lignivorans* SYK-6, a degrader of lignin-based aromatic compounds.^35^ The SYK-6 MTHFR enzyme coevolved with a THF-dependent LigM-type demethylase, and the two enzymes act in concert allowing the bacterium to extract one-carbon unit from lignin-based aromatics for entry into the folate cycle.

## Human MTHFR Has Evolved a Novel Domain and Architecture

It has long been known that human MTHFR (hsMTHFR) harbors more than the CD in the amino acid sequence, encoding a second domain, referred thereafter as the regulatory domain (RD), that is present only in eukaryotic orthologues (Figure 2A).^20,22,23^ The RD structure was revealed for the first time from crystals of hsMTHFR^36^ as a novel fold comprising of two five-stranded β-sheets interspersed with eight α-helices (Figure 3).

**Figure 3:**
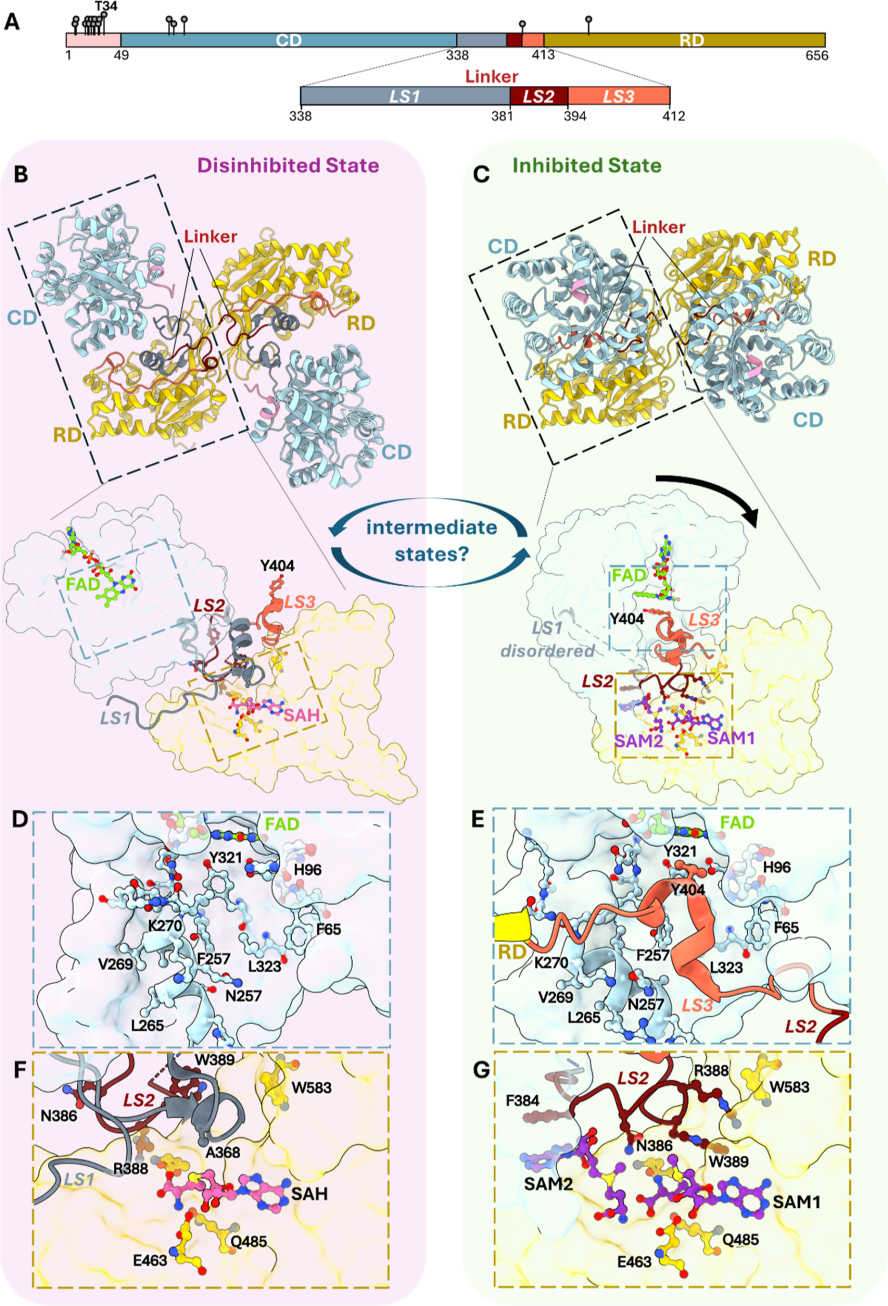
Allosteric regulation of human MTHFR catalysis. (A) Domain organization of hsMTHFR showing phosphorylation sites and highlighting the linker segments. (B–G) Structural basis of hsMTHFR disinhibited and inhibited states, revealing substantial differences in their interdomain orientation (panels B,C), linker conformation (panels B,C inset), active site occupancy (panels D,E), and binding mode of the RD pocket (panels F,G).

At the core of the RD is a binding pocket which was bound with one SAH molecule in the crystal structure, likely copurified with the enzyme during recombinant expression. In the absence of a SAM-bound structure at the time, this pocket was expected to bind SAM in a similar manner to SAH. This assertion was evidenced by their similar chemical structures and supported by solution-binding studies using an RD-alone construct.^37^ As such, the MTHFR RD is topologically distinct from all other SAM-binding folds universally found within the 18 methyltransferase and nonmethyltransferase classes,^38,39^ many of which share a common Rossman fold.^39,40^ The utilization of a unique SAM-binding fold for eukaryotic MTHFR, despite multiple existing ones in Nature, is remarkable. A possible evolutionary origin of this domain was very recently traced back to bacteria that encode proteins of unknown function from the DUF6919 family fold.^41^ Remarkably, DUF6919 members not only have structural homology but also exhibit strictly conserved amino acid positions that map to the RD-binding pocket in hsMTHFR.

In the hsMTHFR structure, the CD and RD are connected via an elaborate interdomain linker that traverses and folds into both domains. HsMTHFR assembles as a homodimer (of 66–70 kDa monomers) in solution, and the structure reveals a dimeric interface formed exclusively via the two RDs of the protomers, such that the CDs are located at opposite ends of the homodimer by the interdomain linker, away from the RD–RD dimer interface (Figures 2D, 3B). Such homodimeric assembly is conserved in eukaryotes, from porcine,^19,20^ plants^23^ down to yeast.^42^

This contrasts with bacteria MTHFRs which lack an RD, and instead, through their CDs exclusively, manifest in a variety of homo-oligomeric states including dimers (e.g., *T. thermophilus* ^43^), tetramers (e.g., *E. coli* ^15^), trimers of homodimers (e.g., *Neisseria meningitidis*) (e.g. ref 16), and octamers (e.g., *Peptostreptococcus productus* ^44^) (Figure 2D). Cooperativity has not been demonstrated for any of these orthologues, suggesting that these multitudinous oligomeric states may exist mainly to maintain the protein stability for catalysis. Interestingly, the recently identified flavin-independent mycobacterial MTHFRs exist as monomers.^30^

## Feedback Regulation of MTHFR in Response to the Cell’s Methylation Potential

MTHFR is a key exemplar of several OCM enzymes where SAM or SAH exerts direct control of enzymatic activity through physical interaction with the enzyme. The cell is highly sensitive to the ratio between SAM and SAH, also known as the cellular methylation potential, due to the great extent of SAM-dependent transmethylation reactions, the majority of which can be inhibited by SAH.^3,45^ Because SAM-dependent methylation regulates key cellular processes, including epigenetic modification, autophagy, and cell cycle progression, the cell invests significant effort in maintaining the SAM/SAH ratio. This includes feed-back regulation of OCM, either via indirect regulation of gene expression or via direct binding to key OCM enzymes.^46^ Relevant to the latter, binding of SAM activates cystathionine β-synthase (CBS) as an allosteric regulator^47,48^ and inhibits the SAM biosynthetic enzyme methionine adenosyltransferase (MAT2A) as its product^49^ (Figure 1). SAM-mediated activation of the CBS enzyme, which diverts homocysteine away from remethylation in the methionine cycle and funnels it into the trans-sulfuration pathway for sulfur metabolism, represents another key decision point in the OCM that is driven by the cellular SAM/SAH ratio.

It was reported in MTHFR that 200 μM SAM is sufficient to nearly abolish its residual activity in purified recombinant enzyme^36^ and endogenously expressed enzyme in HEK293T cells.^37^ This sensitivity is considered a key regulatory feature to prevent depletion of the cytosolic CH_2_-THF pool (required in the folate cycle) for the MTHFR reaction, unless there is a demand for SAM production. Much of what we know about how MTHFR impacts SAM/SAH production has derived from studies conducted in yeast. *S. cerevisiae* harbors two MTHFR isoforms, scMet12 and scMet13.^50^ Absence of scMet13, which shares 43% sequence identity with hsMTHFR and is inhibited by SAM, results in methionine auxotrophy.^22,42,50^ Endogenous expression of a SAM-insensitive chimeric scMet13 results in 140-fold increased accumulation of SAM and 7-fold methionine, with reduced commitment of formate-derived carbon units to SAM synthesis.^22^ Likewise, overexpression of SAM-desensitized (Arg357Ala) scMet13, coupled with methionine supplementation, drives continuous remethylation of homocysteine, resulting in reduced SAH and futile recycling of methionine and SAM.^42^ This ultimately led to depletion of adenine, guanine, and ATP pools and excessive consumption of CH_2_-THF. While these studies may not fully represent the effects of dysregulated MTHFR in humans,^51^ similar disturbances have been found in cultured mouse embryonic fibroblasts,^52^ suggesting a conserved role.

All fungal species and many dicot plants are unique in encoding more than one copy of the *MTHFR* genes (e.g., *S. cerevisiae* *MET12*, *MET13*;^50^ *Schizosaccharomyces pombe* *met9*, *met11*;^53^ *Aspergillus nidulans* *metA*, *metF*;^54^ *A. thaliana* *MTHFR1*, *MTHFR2* ^55^), resulting in different MTHFR isoforms. While multiple MTHFR isozymes in the genome may allow cells to flexibly regulate SAM levels, it is possible that they serve distinct functions, hence requiring them to evolutionarily coexist in these organisms. As exemplified in *S. cerevisiae*, scMet13 being likely the more ancestral isoform based on closer sequence conservation to other eukaryotic orthologues, accounts for the majority of overall MTHFR cellular activity. While deletion of *met13* leads to methionine auxotrophy, deletion of *met12* has no obvious phenotype. Additionally scMet12 overexpression cannot functionally complement the loss of scMet13.^50^ How the two isoforms respond to SAM in their respective functions is currently unknown.

## MTHFR in the Inhibited State

It has long been believed that the RD, unique to eukaryotic MTHFR sequences, would constitute a regulatory element through which SAM exerts its inhibition toward the enzyme’s catalytic activity and through which SAH negates the effect of SAM. The determination of the hsMTHFR crystal structure with an RD pocket occupied by SAH^36^ supports this notion, representing a “disinhibited” state of the enzyme whereby SAH binding physically blocks SAM from enacting its inhibitory role.

This of course implies that the enzyme adopts at least one other conformation, that of an inhibited state in the presence of SAM. In the past year, the SAM-bound structures of MTHFR from human and the thermophilic fungus *Chaetomium thermophilum* (ctMTHFR) have been determined by cryo-electron microscopy and X-ray crystallography, respectively.^56,57^ Comparing the SAH-bound and SAM-bound structures (Figure 3) reveals an extensive interdomain rearrangement between the two structures, realigning the orientation between the CD and RD that results in a ∼30 Å displacement. The SAH-bound (disinhibited) state adopts an “open” conformation in which the two CDs of the homodimer, along with their active sites, are positioned far away from the RDs (Figure 3B).^36^ This contrasts with the SAM-bound (inhibited) state, which is in a more compact “closed” conformation, where CDs are positioned closer to, and face toward the RDs (Figure 3C). Altogether, this constitutes the first structural basis for a two-state model of MTHFR catalysis, first proposed over three decades ago.^10,20^

Looking more closely into the RD pocket, one finds an even bigger surprise. In contrast to the single SAH binding site found in the disinhibited state (Figure 3B, inset), the RD accommodates two SAM molecules in the inhibited state: one SAM occupies the same binding site as SAH, whereas the second binds adjacently in a tail-to-tail manner in a pocket formed only in this state (Figure 3C, inset). Mutations of key residues in either of the two SAM sites of hsMTHFR abolish SAM-mediated inhibition,^56^ indicating that both SAM molecules are essential for inhibition. MTHFR can now be recognized as one of only a handful of proteins known to contain two tandem SAM-binding sites. Others include the *E. coli* radical SAM enzyme coproporphyrinogen III oxidase (HemN) harboring two adjacent but nonequivalent SAM-binding sites, and *A. thaliana* threonine synthase, carrying two symmetrical SAM-binding sites at the dimer interface.^58,59^ As described below, hsMTHFR represents a new example in which the two SAM-binding sites are nonequivalent and assigned different roles.

## Linker-Mediated Transition between Inhibited and Disinhibited States

Although the interdomain orientation changes dramatically between the inhibited and disinhibited states, the overall conformations of the CD and RD themselves remain largely unchanged. So, what brings about the interdomain rearrangement? The key driver is the elaborate linker region, spanning nearly 100 aa, and acting as the potential sensor of the SAM/SAH.

Between the two states, this linker adopts very different conformations and contributes diverse functionalities through its three constituent segments (LS1, LS2, and LS3 in hsMTHFR) (Figure 3A). There is a hinge region (LS1) which stabilizes the disinhibited state by shaping the RD pocket in a way that sterically blocks access of SAM to its second binding site (Figure 3F). In the inhibited state, LS1 moves out into the protein exterior, thereby exposing the second SAM site (Figure 3G). The middle segment (LS2), maintained at the CD–RD interface in the disinhibited state (Figure 3F), moves into the RD in the inhibited state, contributing key interactions to both binding sites of SAM through a conserved F_384_PNGRW_389_ motif (Figure 3G). Site specific substitutions of this motif abrogate SAM binding and inhibition.^56^ The last segment of the linker (LS3) rearranges from being surface exposed in the disinhibited state to becoming buried at the CD–RD interface in the inhibited state. Here, a hydrophobic plug (Tyr404 in hsMTHFR, Tyr361 in ctMTHFR) is translocated over a distance of 30 Å into the CD active site, blocking access of the substrates NADPH/CH_2_-THF (Figure 3E). Similar to the binding mode of the substrates, this inserted tyrosine residue makes stacking interactions with the flavin group of the FAD cofactor (Figure 3C, inset). Thus, in the inhibited state, this plug acts to sterically block substrate access to the active site. This contrasts with the disinhibited state, where access to the CD active site is not restricted (Figure 3D).

It is clear that the linker region has roles beyond connecting the CD and RD. It is the center-piece of a ligand-induced signaling mechanism, relaying to the CD the signal that SAM or SAH has bound the RD in order to mediate an autoinhibitory response at the active site (blocking substrate access to active site). Its key importance is underlined by sequence conservation across eukaryotic orthologues down to *C. thermophilum*, and that it harbors a disproportionately large number of disease-causing variants. With snapshots of the single-SAH-bound disinhibited state and the dual-SAM-bound inhibited state now revealed, the intriguing question is what are the underlying steps needed to induce this transition?

## Are There Intermediate Steps beyond the Two-State Model?

Recognizing that the binding site for SAH (and the first SAM) is positioned deeper into the RD than the binding site for the second SAM, it is reasonable to expect that an intermediate state exists where the RD is bound with only the first SAM molecule. The emerging model therefore posits (at least) two sequential SAM-binding steps: binding of the first SAM to the RD in the disinhibited state, which initiates local rearrangement toward a “poised” intermediate (step 1), which then favors subsequent binding of the second SAM to drive the full transition into the inhibited state (step 2). The implication is that it is the binding of the second SAM in step 2 that results in conformational changes associated with the inhibited state, as described in the previous section. This two-step model could relate to the long-held observation that SAM-mediated hsMTHFR inhibition occurs over the course of minutes, with an initial rapid phase followed by a minutes-long inhibition event upon SAM addition.^10,60^

So how does MTHFR differentiate between the SAH of the disinhibited state and the first SAM (step 1) of the inhibited state, particularly if they bind in the same site? The key could lie in the linker residue Ala368 (in LS1), an invariant position in chordates proposed to be a sensor for the SAM/SAH balance. At low cellular SAM/SAH ratios, MTHFR is expected to be bound to SAH, with Ala368 in close contact (<4 Å) to the SAH homocysteine sulfur (Figure 3F). However, when the SAM/SAH ratio increases, the replacement of SAH with SAM in this site causes a steric clash between Ala368 and the sulfonium methyl group of SAM, forcing rearrangement of the LS1 segment to reposition Ala368, which becomes flexible or disordered in the inhibited state.

This steric relationship between Ala368 and the SAM/SAH sulfonium center is likely the trigger that then cascades into linker rearrangement and domain repositioning, ultimately leading to CD inhibition (step 2). In support of this, substitution of hsMTHFR Ala368 with a larger leucine residue slightly increases SAM sensitivity.^61^ Interestingly, Ala368 can be accommodated by a larger amino acid in lower eukaryotes such as nematodes and fungi (e.g., Arg326 in ctMTHFR), indicating there may be evolutionary differences in how the initial signal is propagated. Further structural studies characterizing putative MTHFR intermediate state(s), including the single SAM-bound state, would facilitate a deeper mechanistic understanding of this mechanism.

## Phosphorylation Status—the Unknown Layer of MTHFR Regulation

SAM-mediated inhibition of MTHFR represents one of a concerted number of ways in which SAM regulates its own synthesis. HsMTHFR can be multiply phosphorylated, mediated via a serine-rich N-terminus (aa 1–47) that is specific to mammals (Figure 2A), and contains 11 out of 16 known phosphorylation sites *in vitro* (Figure 3A).^36,57^ This includes the highly conserved phosphorylation priming site threonine 34, whose substitution to alanine (Thr34Ala) blocks phosphorylation of the entire protein.^62–65^ Several kinases have been implicated in MTHFR phosphorylation, such as cyclin-dependent kinase 1,^64^ polo-like kinase 1,^66^ dual-specificity tyrosine phosphorylation-regulated kinase 1A/2, and glycogen synthase kinase 3A/B.^67^

The phosphorylation status of MTHFR has been shown to impact SAM-mediated inhibition. Recombinant phosphorylated full-length hsMTHFR shows a preference for SAM binding, whereas nonphosphorylated forms, either treated with calf intestine alkaline phosphatase or through a truncated construct lacking the Thr34Ala, show a preference for binding SAH.^36,62^ Importantly, the effect of phosphorylation is clearly evident in kinetic studies, where phosphorylated hsMTHFR shows an inhibitory constant (*K*_i_) of 2.7 μM, compared to the phosphatase-treated enzyme with *K*_i_ of 6.4 μM.^36^ The increased sensitivity for SAM inhibition upon phosphorylation occurs without majorly affecting the overall enzyme activity.^36,62,63^

The underlying mechanism connecting phosphorylation and SAM inhibition is unclear, and limited insight was available from the recent cryo-EM structures due to the dynamic nature of the N-terminus that has eluded visualization. It is possible that the phosphorylation at the N-terminus and other regions of MTHFR would present a series of negative charges that influence the dynamics of the interdomain linker rich in positively charged amino acids. With the linker being the centerpiece for the signaling pathway between CD and RD, phosphorylation could act as a reversible means to fine-tune MTHFR’s sensitivity toward SAM inhibition, in rapid response to ever-changing fluctuations in the cellular methylation status (SAM/SAH ratio). Consistent with this, MTHFR expressed in the presence of high methionine conditions (and indirectly high SAM) shows predominantly phosphorylated protein.^62,67^ HsMTHFR also apparently interacts with the amino acid (including methionine) starvation sensor protein GCN1,^65^ providing a potential link between MTHFR and other cellular methylation-dependent processes, including protein translation.

## MTHFR Link to Health and Disease

Regulation of MTHFR is key for the cell to respond to changes in methylation potential, thereby balancing the acts of the folate and methionine cycles. Dysregulation of MTHFR, due to deficient or defective enzyme, is therefore detrimental in the context of OCM. The cell’s inability to generate CH_3_-THF as the main circulating folate species blocks the remethylation of methionine, leading to low or below-normal levels of methionine and SAM. Disrupted SAM synthesis, and the resultant reduction in cellular methylation capacity, has been shown to perturb myelination and brain development.^68,69^ This blockage of remethylation also builds up homocysteine in the urine and blood,^70^ which is a toxic metabolite when overabundant.^71^ This accumulation causes a diversion of homocysteine into the trans-sulfuration pathway through the CBS enzyme (Figure 1), resulting in elevated cystathionine.^70^

It is therefore not surprising that autosomal recessive pathogenic variants of the *MTHFR* gene lead to severe MTHFR deficiency,^72^ a rare neurological and neurodevelopmental disorder. In 2016, approximately 200 patients were known worldwide, and 109 disease-causing variants were described, the majority (64%) of which are missense changes.^73^ A number of studies have systematically annotated the disease-causing variants toward establishing structure–activity relationships. Enzymatic characterization of patient-derived fibroblast lines demonstrated that the variant residual enzymes have generally below 20% activity of wild-type^74^ or present distinct kinetic abnormalities.^73^ A computational approach^75^ categorized variants based on predictors of the protein–protein interface and free energy changes ΔΔG, taking advantage of the available MTHFR structures. An elegant study^61^ applied yeast multiplexed assays to a library of random mutagenesis of the *MTHFR* gene, to dissect the effects of disease-causing variants under different environmental and genetic backgrounds. Interestingly, certain MTHFR pathogenic variants displayed positive genetic interactions (complementation) with a common single nucleotide polymorphism p.Ala222Val^74,76,77^ and with dietary folate.

MTHFR has also been exploited for the development of novel therapeutics, for example, toward cancer treatment. A significant upregulation of MTHFR expression has been seen in prostate tumor tissue,^78^ indicating its role in supporting increased metabolic demands of cancer cells. Antisense inhibition of MTHFR has been shown to reduce tumor growth of lung and colon cancer cell lines *in vitro* and xenografts *in vivo*.^79,80^ Co-administration of MTHFR with 5-fluorouracil has also shown synergistic effects on tumor growth reduction.^79^ These studies from nearly two decades ago have not been followed up further in disease models. Recent meta-analyses of mRNA expression in human tumors^81^ have also not identified MTHFR as a widely applicable cancer target.

The knowledge gained from the repertoire of MTHFR structures from humans to bacteria will enlighten the next generation of small-molecule drug discovery. For example, the elucidation of two different ligand-induced conformations in hsMTHFR means that one could envisage designing SAM-like molecules targeting the inhibited state as indication for MTHFR overexpression, or SAH-like molecules targeting the active site as indication for MTHFR deficiency.^37^ Added to this, exploiting structural and sequence differences between species, as exemplified by the remodelled active site in *M. smegmatis* MSMEG_6649 (e.g., featuring a unique NADH site compared to other bacterial MTHFRs),^31^ could provide a path toward developing novel and selective antimycobacterial agents.^82^

## Conclusion

Found in all domains of life, MTHFR catalyzes an evolutionarily preserved reaction that balances the acts of folate and methionine cycles to generate the universal one-carbon precursors for a series of anabolic pathways. Yet this age-old enzyme has so elegantly tailored features in cofactor preference, oligomeric status, as well as allosteric regulation to suit an organism’s unique and specific metabolic needs. The past decade has witnessed a huge leap forward in our understanding of the enzyme’s biochemistry, thanks to structural biology and complementary approaches. The next decade promises to be exciting too, with potential discovery of novel functional partners, crosstalk regulations, and target-based therapeutics that continue to define the intricate biology of MTHFR. In the era of cutting-edge proteomics, metabolomics, and chemical biology, we look forward to new research in MTHFR that unlocks future horizons toward better human health.
